# Anticoagulation optimization and clinical impact of post-transplant deep vein thrombosis: clinical impact and a VV-ECMO subgroup analysis

**DOI:** 10.1007/s10047-026-01554-x

**Published:** 2026-05-05

**Authors:** Chitaru Kurihara, Yudai Miyashita, Taisuke Kaiho, Dai Yamanouchi

**Affiliations:** 1 Division of Thoracic Surgery, Department of Surgery, Northwestern University Feinberg School of Medicine, 676 N. Saint Clair St., Suite 650, Chicago, IL 60611 USA; 2 Department of Vascular Surgery, Fujita Health University, Toyoake, Japan

**Keywords:** Lung transplantation, Deep venous thrombosis, Pulmonary embolism, Extracorporeal membrane oxygenation

## Abstract

Post-transplant deep vein thrombosis (DVT) is common after lung transplantation and may contribute to pulmonary embolism and other complications. Whether DVT adversely affects contemporary outcomes and how venovenous ECMO (VV-ECMO) bridging influences early DVT patterns remain unclear. We performed a retrospective single-center study of adult lung transplant recipients (2018–2025). DVT and clinical outcomes were ascertained. Primary analyses compared outcomes and overall survival between recipients with and without DVT in the overall cohort. Secondary analyses assessed DVT-free survival and early anatomic distribution among recipients bridged with preoperative VV-ECMO. Comparisons used Fisher’s exact/Mann–Whitney U tests; survival used Kaplan–Meier/log-rank tests. Among 502 recipients, 240 developed DVT. Compared with those without DVT, recipients with DVT had higher rates of pulmonary embolism (22.5% vs. 5.0%, p < 0.0001), acute kidney injury (52.9% vs. 41.2%, p = 0.009), PGD grade 3 (18.3% vs. 10.7%, p = 0.016), longer hospitalization (21 vs. 14 days, p < 0.001), and worse overall survival (HR 2.19, 95% CI 1.49–3.23; log-rank p < 0.001). Of 240 DVT cases, 31 (12.9%) were bridged with VV-ECMO. Within 14 days, upper-extremity DVT was more frequent with VV-ECMO (53.3% vs. 23.5%, p = 0.03), whereas lower-extremity and neck distributions were similar. In multivariable models, operative time was independently associated with DVT (OR 1.18 per hour, 95% CI 1.07–1.31, p < 0.001). Post-transplant DVT is frequent and portends worse outcomes. VV-ECMO bridging is associated with an upper-extremity–predominant DVT pattern but is not an independent DVT predictor after adjustment. Vigilant surveillance is warranted in this high-risk population.

**Supplementary Information:**

The online version contains supplementary material available at 10.1007/s10047-026-01554-x.

## Introduction

Venous thromboembolism (VTE), encompassing deep vein thrombosis (DVT) and pulmonary embolism, remains a frequent and serious complication among critically ill patients [1]. Lung transplant recipients are particularly predisposed to thrombotic events owing to multiple interacting factors, including systemic inflammation, endothelial activation, and immobility during prolonged hospitalization [2]. The increasing use of with veno-venous extracorporeal membrane oxygenation (VV-ECMO) as a bridge to lung transplantation has markedly improved the survival of patients with end-stage lung disease; however, it introduces complex alterations in hemostasis that predispose to both thrombosis and bleeding [3–5]. The VV-ECMO circuit itself activates the coagulation cascade and platelets, while systemic anticoagulation, necessary to prevent circuit clotting, simultaneously increases bleeding risk [6–8]. Thus, managing the delicate balance between thrombosis prevention and hemorrhage remains a critical and unresolved challenge in this high-risk population.

During VV-ECMO support, the pathogenesis of DVT is multifactorial, involving venous stasis distal to cannulation sites, contact activation from the artificial circuit, and systemic inflammatory responses. Furthermore, many cases remain clinically silent, detectable only through systematic screening, underscoring the underrecognized burden of thrombosis in VV-ECMO-supported patients. Despite improvements in circuit design and anticoagulation protocols, DVT remains a persistent problem in those bridged to lung transplantation. After successful transplantation, these patients enter a unique hemostatic milieu characterized by surgical trauma, immobilization, transfusion-related coagulopathy, and evolving inflammatory and immunologic changes. The early post-transplant period, in particular, represents a vulnerable window during which both bleeding and thrombosis are common. Immunosuppressive therapy and infection can further modulate coagulation pathways, amplifying thrombotic risk. Although the focus of prior research has been primarily on bleeding complications or allograft dysfunction, thromboembolic complications after transplantation—especially in patients previously supported with VV-ECMO—have received limited systematic evaluation. Several studies have described thrombotic events during VV-ECMO support [9–13], but only a few have addressed their occurrence and impact after transplantation. While anticoagulation management is central to preventing thrombosis, strategies differ widely among institutions, reflecting uncertainty regarding optimal timing and intensity of therapy resumption following transplantation. International consensus guidance has increasingly standardized key aspects of VV-ECMO management, including patient selection, cannulation strategy, and circuit management. However, anticoagulation practices remain heterogeneous across centers, with substantial inter-institutional variation in monitoring assays (ACT, aPTT, anti-Xa) and target ranges [14, 15]. Balancing the risks of postoperative bleeding, graft dysfunction, and thrombosis remains one of the most challenging aspects of peri-transplant management. Consequently, critical gaps persist in understanding the incidence, risk factors, and clinical consequences of post-transplant DVT in VV-ECMO-bridged recipients. It remains unclear whether DVT after transplantation influences early recovery, intensive care unit (ICU) stay, mechanical ventilation duration, or longer-term outcomes such as graft function and survival. Similarly, the impact of varying anticoagulation strategies on the development and progression of DVT in this population has not been defined. Addressing these questions is essential, as identification of modifiable factors could directly inform perioperative management and improve both short- and long-term transplant outcomes. At our center, VV-ECMO bridging is routinely managed using a heparin-sparing approach in which continuous systemic anticoagulation is withheld unless clinically indicated such as DVT/PE, an approach that has been reported to be feasible without excess major thrombotic complications in selected VV-ECMO patients [16]. In this retrospective single-center study, the primary objective was to define the incidence and clinical impact of post-transplant DVT in the overall lung transplant cohort. Secondary objectives were to (i) characterize DVT risk and early anatomic distribution in recipients bridged with preoperative VV-ECMO and (ii) compare DVT-free survival by VV-ECMO bridging status. Exploratory analyses evaluated anticoagulation patterns in relation to thromboembolic and hemorrhagic outcomes. Together, these analyses aim to inform peri-transplant thrombosis surveillance and anticoagulation decision-making.

## Patients and methods

### Study design

This retrospective single-center study was conducted at Northwestern University Medical Center in Chicago, Illinois, USA. The study protocol was approved by the Institutional Review Board of Northwestern University (STU00207250 and STU00213616). The need for informed consent was waived due to the retrospective nature of the study. Adult patients (≥ 18 years old) who underwent lung transplantation at Northwestern University between January 2018 and April 2025 were eligible for inclusion. Patients who received multiorgan transplants or underwent retransplantation were excluded. Clinical data were retrieved from institutional electronic medical records and entered into a prospectively maintained lung transplant database. This study focused on patients bridged to lung transplantation with preoperative VV-ECMO. Patients supported with veno-arterial ECMO (VA-ECMO) or VAV-ECMO as a preoperative bridge were excluded from the analytic cohort. Intraoperative VA-ECMO use for cardiopulmonary support during transplantation was recorded as an operative variable and does not represent the preoperative bridging ECMO modality. Comprehensive data were collected on recipient demographics, baseline comorbidities, donor characteristics, preoperative laboratory results, and intraoperative variables including ischemic time, use and duration of VV-ECMO support, and transfusion requirements. Postoperative outcomes were also recorded, including duration of mechanical ventilation, intensive care unit (ICU) length of stay, hospital stay, and early complications. The primary outcome was development of post-transplant DVT. The primary analysis evaluated the association of DVT with postoperative outcomes and survival; secondary analyses evaluated DVT patterns and outcomes in the VV-ECMO–bridged subgroup. Secondary outcomes included ICU length of stay, postoperative bleeding events, need for reoperation, 30-day and 1-year mortality, and overall survival. The association between post-transplant DVT and clinical outcomes was analyzed.

### Perioperative VV-ECMO indication criteria

Prior to lung transplantation, all intubated patients were treated by a multidisciplinary team in accordance with the guidelines of the National Heart, Lung, and Blood Institute’s ARDS Network [17]. Indications for VV-ECMO evaluation included refractory hypoxemia with PaO_2_ less than 55 mmHg, pulse oximetry oxygen saturation less than 88%, and pH level less than 7.2. Patients were evaluated with lung-protective mechanical ventilation with a plateau pressure of less than 35 mmHg, neuromuscular blockade, and prone positioning, according to recommendations from the Extracorporeal Life Support Organization [18].

### Anticoagulation during VV-ECMO support

Patients did not receive continuous anticoagulation unless there was a specific indication, such as deep venous thrombosis or pulmonary embolism, and there was no monitoring of bleeding parameters, such as activated clotting time or activated partial thromboplastin time. All patients who were not receiving continuous systemic anticoagulation received 5,000 U of subcutaneous unfractionated heparin every 8 h as a prophylactic dose to prevent deep venous thrombosis. VV-ECMO flow was maintained at a minimum of 3.0–3.5 L/min, consistent with our recent reports, to reduce thrombotic complications in the VV-ECMO circuit [16, 19–21]. In this study, “anticoagulation use” was defined as receipt of systemic anticoagulation at any time after lung transplantation (e.g., intravenous unfractionated heparin infusion and/or subsequent transition to an oral anticoagulant), initiated for clinical indications such as DVT or PE at the discretion of the treating team.

### DVT surveillance and imaging

Venous duplex ultrasonography for DVT assessment was not performed as routine screening after lung transplantation at our institution. Instead, duplex imaging was obtained only when clinically indicated based on symptoms or provider concern (e.g., limb swelling or pain, unexplained hypoxemia, or other findings suggestive of thromboembolism) [22]. Because duplex examinations were ordered on an as-needed basis, the number and timing of duplex studies were not captured in a uniform structured format in the dataset and could not be reliably quantified across patient subgroups.

### Definition of complication

#### Primary graft dysfunction (PGD)

PGD was defined based on the ISHLT guideline and graded by PaO2/FiO2 ratio as follows; Grade 1: PaO2/FiO2 ratio > 300; Grade 2: PaO2/FiO2 ratio is 200–300; Grade 3: PaO2/FiO2 ratio < 200 [23].

#### Chronic lung allograft dysfunction (CLAD)

CLAD is defined as a sustained (at least 3 months) decline in the forced expiratory volume in one second (FEV₁) of at least 20% from the post-transplant baseline, in the absence of other reversible causes. CLAD is further sub-classified into phenotypes such as bronchiolitis obliterans syndrome (BOS) and restrictive allograft syndrome (RAS) based on clinical, radiologic, and physiologic criteria [24].

#### Acute kidney injury (AKI)

AKI was defined using the Risk, Failure, Loss of kidney function, and End-stage kidney disease classification [25].

### Statistical analysis

Continuous variables were expressed as median (interquartile range, IQR) and compared using the Mann–Whitney U test. Categorical variables were expressed as counts and percentages and compared using Fisher’s exact test. Survival curves were estimated using the Kaplan–Meier method and compared using the log-rank test. Univariate and multivariate logistic regression analyses were used to identify predictors of post-transplant DVT, whereas Cox proportional hazards analysis was used to evaluate the effect of DVT on mortality. Variables with a *p*-value < 0.10 in univariate analysis were included in multivariate models. Statistical significance was defined as *p* < 0.05. All analyses were performed in R (version 4.2.1) using the survival and survminer packages.

## Results

### Patient characteristics

A total of 502 lung transplant recipients were included (240 with postoperative DVT and 262 without). Baseline characteristics were broadly comparable between groups (Supplementary Table 1). Pre-transplant VV-ECMO bridging was more frequent in the DVT group (12.9% vs. 8.4%), though this did not reach statistical significance (p = 0.11). Pulmonary arterial hypertension was less common among patients with DVT (5.0% vs. 10.3%, p = 0.03). Intraoperative parameters were similar, except for longer operative time in the DVT group (5.6 [4.6–7.4] vs. 5.1 [3.9–6.6] hours, p = 0.01). Postoperatively, DVT was associated with higher rates of PE (22.5% vs. 5.0%, p < 0.0001), AKI (p = 0.009), and PGD grade 3 (p = 0.016), as well as greater resource use including hemodialysis after discharge, longer hospital stay, and more frequent post- VV-ECMO utilization (all p ≤ 0.010).

### Survival outcomes

**Fig. 1:**
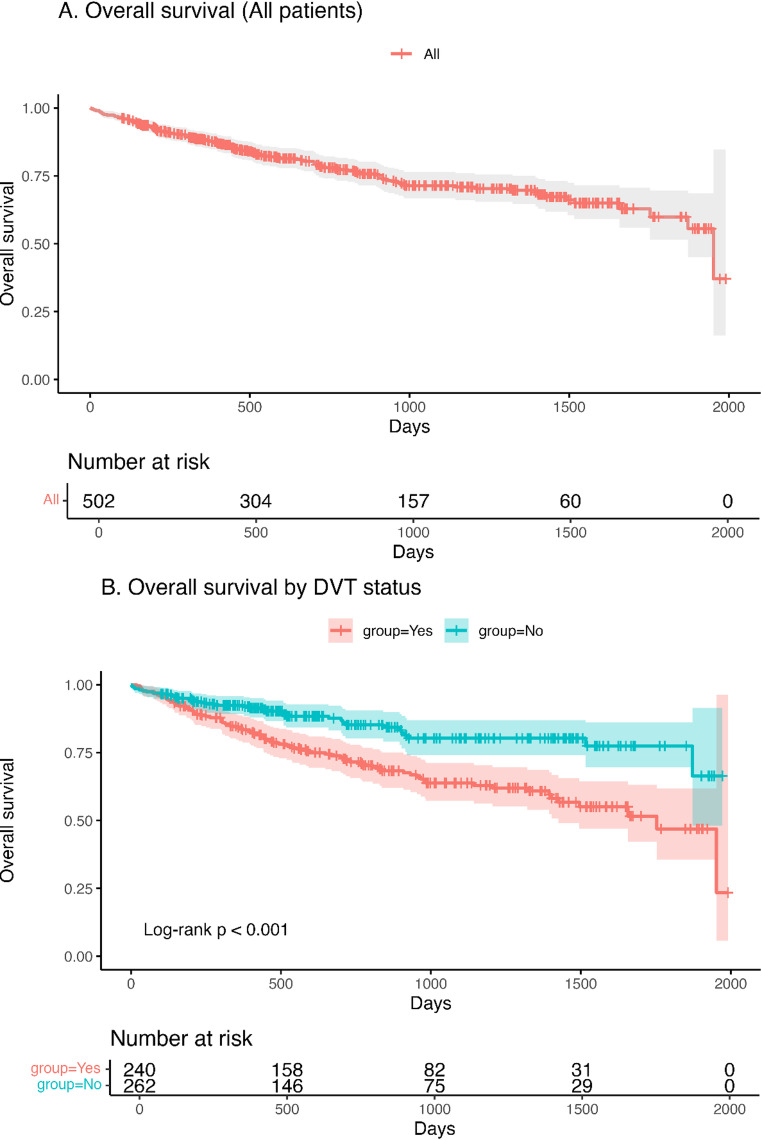
Overall survival after lung transplantation. **A** Kaplan–Meier curve showing overall survival of all patients after lung transplantation. **B** Overall survival stratified by the presence or absence of postoperative deep vein thrombosis (DVT)

Kaplan–Meier analysis demonstrated significantly lower overall survival among patients with DVT compared to those without DVT (log-rank *p* < 0.001; HR 2.19, 95% CI 1.49–3.23; Fig. 1). One-, three-, and five-year survival was 83.8%, 63.8%, and 46.9% in the DVT group versus 92.4%, 80.3%, and 77.4% without DVT. The DVT cohort showed a clear separation of survival curves early after transplantation, and the difference persisted throughout the observation period.

### Impact of VV-ECMO use on clinical characteristics in the DVT cohort

Among the 240 patients with postoperative DVT, 31 (12.9%) were bridged with pre-transplant VV-ECMO (Supplementary Table 2). Compared with DVT patients without preoperative ECMO, VV-ECMO–bridged patients were younger and underwent more complex operations, with longer operative time and substantially higher transfusion requirements. Postoperatively, they had higher rates of anticoagulation use, PGD grade 3, and dialysis, and experienced prolonged ventilator support and hospitalization (all p < 0.05).

### Distribution of DVT locations according to VV-ECMO use

Among patients who developed DVT within 14 days, upper-extremity (UE) DVTs were more frequent in the pre-operative VV-ECMO group than in the non- VV-ECMO group (53.3% [8/15] vs. 23.5% [19/81]; p = 0.03). In contrast, the proportions of lower-extremity DVT above the knee (20.0% vs. 23.5%; p = 1.00) and below the knee (40.0% vs. 43.2%; p = 1.00), as well as neck-vein thrombosis (26.7% vs. 45.7%; p = 0.26), did not differ significantly between groups. Within each anatomical stratum, rates of therapeutic anticoagulation were similar in VV-ECMO and non- VV-ECMO patients (Table 1, Supplementary Table 3).

**Table 1:** DVT locations within 14 days post-transplant, stratified by preoperative VV-ECMO use, with anticoagulation by location

Location	Preoperative VV-ECMO use			Anticoagulation		
	VV-ECMO (n = 15)	No VV-ECMO (n = 81)	p-value	VV-ECMO	No VV-ECMO	p-value
Upper extremity	8 (53.3%)	19 (23.5%)	0.03	6/8 (75.0%)	15/19 (78.9%)	1.00
Lower extremity above knee	3 (20.0%)	19 (23.5%)	1.00	3/3 (100.0%)	13/19 (68.4%)	0.53
Lower extremity below knee	6 (40.0%)	35 (43.2%)	1.00	6/6 (100.0%)	26/35 (54.3%)	0.31
Neck vein	4 (26.7%)	37 (45.7%)	0.26	2/4 (50.0%)	26/37 (70.3%)	0.58

DVT, deep vein thrombosis; ECMO, extracorporeal membrane oxygenation; VV-ECMO, venovenous extracorporeal membrane oxygenation. Percentages are based on all DVT cases within each group. Because subcategories are not mutually exclusive, parent and child percentages are not additive. Several entries are duplicate listings across anatomical blocks. P values are from two-sided Fisher’s exact tests

### Risk factors for postoperative DVT

Univariate logistic regression identified several perioperative factors associated with the development of DVT after lung transplantation (Table 2). Among intraoperative parameters, longer operative time was significantly associated with an increased risk of DVT (odds ratio [OR] = 1.18, 95% CI 1.08–1.29, p < 0.001). Postoperative AKI (OR = 1.60, 95% CI 1.13–2.28, p = 0.009), PGD grade 3 (OR = 1.88, 95% CI 1.13–3.15, p = 0.02), and post- VV-ECMO use (OR = 2.08, 95% CI 1.21–3.64, p = 0.009) were also significantly correlated with DVT occurrence. Among preoperative variables, PAH showed a negative association with DVT risk (OR = 0.42, 95% CI 0.19–0.88, p = 0.03).

**Table 2:** Univariate and multivariate logistic regression analysis to predict DVT

Variable	Univariate			Multivariate		
	OR	95% CI	P value	OR	95% CI	P value
*Pre-operative characteristics*						
Age, years	1.01	1.00–1.03	0.08			
*Sex*						
Male	1.19	0.83–1.69	0.34			
BMI, kg/m2	1.01	0.97–1.05	0.77			
BSA, m2	1.43	0.70–2.92	0.33			
*Smoking history*						
Yes	0.90	0.63–1.28	0.56			
Hypertension	1.06	0.75–1.52	0.73			
Diabetes	1.13	0.77–1.66	0.53			
CKD	1.47	0.80–2.74	0.22			
Dialysis	1.44	0.62–3.44	0.39			
On the waiting list (days)	1.00	1.00–1.01	0.21			
Preoperative VV-ECMO	1.62	0.91–2.91	0.10	0.76	0.36–1.58	0.47
*Etiology*						
ILD	0.84	0.55–1.30	0.44			
COPD	0.98	0.57–1.67	0.93			
PAH	0.42	0.19–0.88	0.03	0.41	0.19–0.83	0.02
Others	1.00	Reference				
*Laboratory*						
Hemoglobin, g/dL	0.93	0.87–1.00	0.06			
Platelets, 1,000/mm3	1.00	1.00–1.00	0.23			
Creatinine, mg/dL	1.05	0.98–1.49	0.60			
INR	0.98	0.36–2.64	0.96			
PTT	0.99	0.98–1.00	0.29			
PRA	1.10	0.77–1.57	0.61			
*Intra-operative outcomes*						
Bilateral	1.04	0.72–1.50	0.84			
Operative time (hours)	1.18	1.08–1.29	< 0.001	1.18	1.07–1.31	< 0.001
Intra-op blood transfusion; pRBC	1.04	0.99–1.09	0.14			
Intra-op blood transfusion; FFP	1.07	0.98–1.17	0.14			
Intra-op blood transfusion; Plt	1.14	0.98–1.35	0.10			
VA ECMO use	1.13	0.79–1.63	0.49			
*Post-operative outcomes*						
AKI	1.6	1.13–2.28	0.009	1.43	0.98–2.08	0.06
PGD grade3	1.88	1.13–3.15	0.02	1.44	0.80–2.61	0.22
Dialysis	1.44	0.62–3.44	0.39			
Post-ECMO use	2.08	1.21–3.64	0.009	1.28	0.63–2.63	0.50

AKI, acute kidney injury; BSA, body surface area; BMI, body mass index; CI, confidence interval; CKD, chronic kidney disease; COPD, chronic obstructive pulmonary disease; DVT, deep vein thrombosis; ECMO, extracorporeal membrane oxygenation; FFP, fresh frozen plasma; ILD, interstitial lung disease; INR, international normalized ratio; OR, odds ratio; PAH, pulmonary arterial hypertension; PGD, primary graft dysfunction; Plt, platelets; PRA, panel reactive antibody; pRBC, packed red blood cells; PTT, partial thromboplastin time; VA-ECMO, venoarterial extracorporeal membrane oxygenation; VV-ECMO, venovenous extracorporeal membrane oxygenation

OR and 95% CI were calculated using logistic regression models. Variables with P < 0.10 in univariate analysis were included in the multivariate model

On multivariate logistic regression, operative time remained an independent predictor of DVT (OR = 1.18, 95% CI 1.07–1.31, p < 0.001). In contrast, preoperative PAH continued to demonstrate a protective association (OR = 0.41, 95% CI 0.19–0.83, p = 0.02). Although postoperative AKI (OR = 1.43, 95% CI 0.98–2.08, p = 0.06) showed a trend toward significance, it did not reach statistical significance in the multivariate model. Other perioperative factors, including preoperative VV-ECMO bridging, post- VV-ECMO use, and PGD grade 3, were not independently associated with DVT after adjustment for confounders.

### Impact of VV-ECMO use on DVT-free survival

**Fig. 2:**
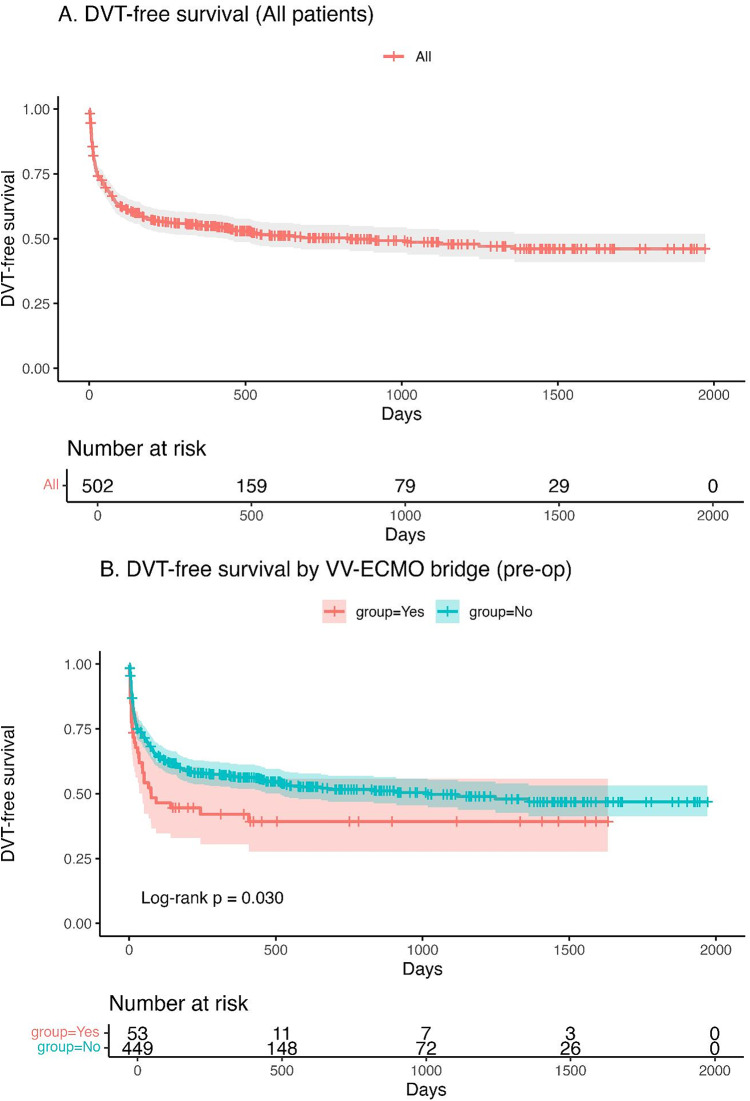
DVT-free survival after lung transplantation. **A** DVT-free survival in the entire cohort. **B** DVT-free survival stratified by preoperative VV-ECMO bridge

The cumulative DVT-free survival of all 502 lung transplant recipients is shown in Fig. 2A. The majority of DVT events occurred within the early postoperative period, with the survival curve plateauing after approximately 1,000 days. Patients bridged to transplantation with VV-ECMO demonstrated significantly lower DVT-free survival compared with those without preoperative VV-ECMO support (log-rank *p* = 0.030; Fig. 2B).

### VV-ECMO–bridged subgroup analysis

Among VV-ECMO–bridged lung transplant recipients (n = 53), 31 developed postoperative DVT and 22 did not. Baseline and intraoperative characteristics were largely comparable between groups; however, patients who developed DVT had longer time on the waitlist and longer VV-ECMO bridge duration (Table 3). Postoperative outcomes were also broadly similar, although PE occurred numerically more often in the DVT group (22.6% vs. 4.5%), without reaching statistical significance in this small subgroup (Table 4). We further examined the relationship between anticoagulation exposure and subsequent PE within the VV-ECMO cohort (Supplementary Fig. 1). Case-level review confirmed that no patients-initiated anticoagulation after PE diagnosis; anticoagulation exposure, when present, occurred before the PE event (Supplementary Table 4).

**Table 3:** Baseline and intraoperative characteristics of VV-ECMO–bridged lung transplant recipients with and without post-transplant DVT

Variable	DVT (n = 31)	No DVT (n = 22)	*P* value
*Pre-operative characteristics*			
Age, years	53.0 (43.5, 58.5)	44.5 (34.0, 53.5)	0.05
Sex			1.00
Male	16/31 (51.6%)	12/22 (54.5%)	
Female	15/31 (48.4%)	10/22 (45.5%)	
BMI, kg/m2	26.1 (22.5, 28.6)	26.6 (22.0, 29.5)	0.84
BSA, m2	1.9 (1.7, 2.1)	1.8 (1.7, 2.0)	0.42
Smoking history			0.20
Yes	5/31 (16.1%)	7/22 (31.8%)	
No	26/31 (83.9%)	15/22 (68.2%)	
Hypertension	15/31 (48.4%)	9/22 (40.9%)	0.78
Diabetes	6/31 (19.4%)	8/22 (36.4%)	0.21
CKD	1/31 (3.2%)	2/22 (9.1%)	0.56
Dialysis	6/31 (19.4%)	2/22 (9.1%)	0.45
On the waiting list (days)	7.0 (4.0, 24.5)	4.0 (3.0, 6.0)	0.04
VV ECMO bridge duration	42.0 (24.0, 113.5)	14.0 (5.5, 59.5)	0.03
*Etiology*			
ILD	9/31 (29.0%)	7/22 (31.8%)	1.00
COPD	0/31 (0.0%)	0/22 (0.0%)	1.00
PAH	1/31 (3.2%)	2/22 (9.1%)	0.56
Others	21/31 (67.7%)	13/22 (59.1%)	0.57
*Laboratory*			
Hemoglobin, g/dL	7.5 (7.2, 8.5)	8.2 (7.6, 9.0)	0.18
Platelets, 1,000/mm3	151.0 (103.0, 197.5)	141.5 (122.0, 214.8)	1.00
Creatinine, mg/dL	0.6 (0.4, 0.8)	0.6 (0.4, 0.9)	0.97
INR	1.2 (1.1, 1.3)	1.1 (1.1, 1.3)	0.55
PTT	34.3 (29.9, 38.2)	33.7 (30.8, 37.8)	0.94
PRA	1.0 (0.0, 1.0)	0.5 (0.0, 1.0)	0.42
*Intra-operative outcomes*			
Bilateral	30/31 (96.8%)	22/22 (100.0%)	1.00
Operative time (hours)	8.2 (6.7, 9.6)	7.3 (5.4, 9.0)	0.15
Intra-op blood transfusion; pRBC	7.0 (5.0, 11.5)	8.0 (6.0, 13.2)	0.79
Intra-op blood transfusion; FFP	3.0 (1.5, 6.0)	2.0 (1.0, 6.0)	0.58
Intra-op blood transfusion; Plt	2.0 (1.0, 3.5)	2.0 (1.0, 3.8)	0.94
VA ECMO use	30/31 (96.8%)	20/22 (90.9%)	0.56
VA ECMO time (hours)	3.1 (2.6, 4.2)	3.5 (2.7, 4.0)	0.68

Continuous variables are presented as median (interquartile range [IQR]); categorical variables are presented as n/N (%). Group comparisons were performed using the Wilcoxon rank-sum test for continuous variables and Fisher’s exact test for categorical variables. VV-ECMO bridge duration is reported in days; VA-ECMO time is reported in hours. BMI, body mass index; BSA, body surface area; CKD, chronic kidney disease; COPD, chronic obstructive pulmonary disease; ECMO, extracorporeal membrane oxygenation; ILD, interstitial lung disease; INR, international normalized ratio; PAH, pulmonary arterial hypertension; PRA, panel reactive antibody; PTT, partial thromboplastin time; VV, venovenous; VA, venoarterial

**Table 4:** Postoperative outcomes of VV-ECMO–bridged lung transplant recipients with and without post-transplant DVT

Variable	DVT (n = 31)	No DVT (n = 22)	*P* value
*Post-operative outcomes*			
de novo DSA	7/31 (22.6%)	6/22 (27.3%)	0.75
CVA	0/31 (0.0%)	1/22 (4.5%)	0.42
Bowel Ischemia	0/31 (0.0%)	1/22 (4.5%)	0.42
Digital Ischemia	2/31 (6.5%)	3/22 (13.6%)	0.64
Days of DVT after lung txplt	18.0 (6.0, 51.0)	–	
PE	7/31 (22.6%)	1/22 (4.5%)	0.12
Days of PE after lung txplt	56.0 (5.5, 70.0)	6.0 (6.0, 6.0)	
Anticoagulation	19/31 (61.3%)	11/22 (50.0%)	0.57
AKI	20/31 (64.5%)	15/22 (68.2%)	1.000
PGD grade3	14/31 (45.2%)	10/22 (45.5%)	1.000
Dialysis	11/31 (35.5%)	5/22 (22.7%)	0.38
HD after discharge	10/31 (32.3%)	2/22 (9.1%)	0.09
Post transplant ventilator	3.0 (1.5, 13.5)	10.0 (3.0, 16.5)	0.12
Hospital stay	35.0 (25.0, 44.0)	37.5 (24.2, 46.2)	0.73
post ECMO use	21/31 (67.7%)	13/22 (59.1%)	0.570

Continuous variables are presented as median (interquartile range [IQR]); categorical variables are presented as n/N (%). Group comparisons were performed using the Wilcoxon rank-sum test for continuous variables and Fisher’s exact test for categorical variables. “Days of DVT after transplant” and “Days of PE after transplant” are calculated among patients who developed the event. No p value is provided due to small event counts. AKI, acute kidney injury; CVA, cerebrovascular accident; DSA, donor-specific antibody; ECMO, extracorporeal membrane oxygenation; HD, hemodialysis; PE, pulmonary embolism; PGD, primary graft dysfunction; VV, venovenous

### Association between VV-ECMO Use, anticoagulation, and thromboembolic events

In the analysis of anticoagulation patterns stratified by VV-ECMO status, DVT, and PE (Supplementary Table 5), anticoagulation therapy was administered more frequently in patients who developed thromboembolic complications and in those requiring VV-ECMO support. Among patients supported with preoperative VV-ECMO, anticoagulation was used in 50.0% of those with both DVT and PE and 64.0% of those with DVT alone, compared with 68.0% and 35.3%, respectively, in patients without VV-ECMO. Overall, anticoagulation utilization was lowest among patients without DVT or PE who did not require VV-ECMO.

In the subgroup of patients who developed DVT, the relationship between anticoagulation and PE was further examined (Supplementary Fig. 2). The incidence of PE was significantly higher in patients who received anticoagulation than in those who did not (49/180 (27.2%) vs. 5/60 (8.3%), p = 0.002).

Consistent with this finding, Kaplan–Meier analysis of PE-free survival (Supplementary Fig. 3) showed that patients receiving anticoagulation had significantly worse PE-free survival compared with those not receiving anticoagulation (log-rank p = 0.0053). The difference between the two groups became evident early after transplantation and persisted throughout follow-up.

### Relationship between anticoagulation and hemorrhagic complications

To assess whether anticoagulation increased the risk of bleeding, hemothorax and hematoma-free survival was compared between patients who did and did not receive anticoagulation (Supplementary Fig. 4). There was no significant difference in hemothorax/hematoma-free survival between the two groups (log-rank p = 0.367). Both curves showed a modest early decline, but long-term survival remained comparable thereafter.

## Discussion

In this single-center retrospective analysis, we identified a high incidence of post-transplant DVT among lung transplant recipients and demonstrated that DVT was associated with significantly worse postoperative outcomes, including higher rates of pulmonary embolism, acute kidney injury, PGD grade 3, and prolonged hospitalization. Importantly, overall survival was significantly reduced in recipients who developed DVT compared with those who did not. Preoperative VV-ECMO was not independently associated with postoperative DVT after multivariable adjustment; however, VV-ECMO bridging identified a high-risk clinical phenotype, characterized by greater perioperative complexity and higher early post-transplant thrombotic hazard, supporting the need for intensified early surveillance in this subgroup. Together, these findings underscore the clinical relevance of thromboembolic complications in lung transplantation and highlight the need for optimized anticoagulation strategies, particularly for patients requiring VV-ECMO support as a bridge to transplant. Notably, VV-ECMO bridging has emerged as a standard practice for patients with acute or rapidly progressive respiratory failure awaiting transplantation [4, 26–29]. Although it improves pretransplant survival, VV-ECMO introduces complex hemostatic alterations due to continuous exposure of blood to artificial surfaces, systemic inflammation, and fluctuating anticoagulation levels [30]. Prior investigations have shown that DVT can occur in up to 40–50% of VV-ECMO-supported patients [30–32]. In our study, 12.9% of patients with post-transplant DVT had preoperative VV-ECMO support, and these patients exhibited more severe perioperative coagulopathy, longer operative times, and higher transfusion requirements. The association between DVT and adverse outcomes may be multifactorial. One can be that the observed association between DVT and acute kidney injury suggests a broader prothrombotic systemic milieu, possibly mediated by venous congestion, systemic inflammation, and impaired microcirculation [30].

The observed inverse association between preoperative PAH and post-transplant DVT deserves cautious interpretation. Although PAH was independently associated with lower odds of DVT in our multivariable model, the number of PAH recipients in this cohort was relatively small, and this finding may be susceptible to residual confounding or model instability. Clinically, several non-mutually exclusive explanations are possible. PAH recipients may undergo more standardized hemodynamic management and early postoperative monitoring, and their perioperative care pathways may differ with respect to fluid management, mobilization, and thresholds for initiating pharmacologic prophylaxis or therapeutic anticoagulation. In addition, recipient selection and underlying disease biology may differ from ILD/COPD populations in ways that are not fully captured by the available covariates. Therefore, this association should be considered hypothesis-generating and warrants validation in larger, multicenter datasets with more granular perioperative management variables.

The relationship between anticoagulation therapy and pulmonary embolism warrants special consideration. The higher rate of PE among anticoagulated patients likely reflects treatment bias—patients with more extensive or symptomatic DVT were preferentially anticoagulated rather than anticoagulation being causative of PE. Importantly, anticoagulation was not associated with a higher incidence of hemorrhagic complications, suggesting that appropriately titrated therapy may be safe even in the early post-transplant setting. This finding is clinically relevant, as concerns about postoperative bleeding often delay or limit anticoagulation initiation in this population. Lastly, comparing VV-ECMO bridge and non VV-ECMO bridge group, there was no significant difference in the day of development DVT after lung transplant. Therefore, screening timing of DVT could be similar in both groups. Consistent with Fig. 2b, the VV-ECMO–bridged cohort shows a higher early hazard of DVT than non-bridged recipients, underscoring the need for intensified early postoperative surveillance and a lower threshold for diagnostic imaging.

These results emphasize the need for proactive DVT surveillance and individualized anticoagulation management in VV- ECMO bridged lung transplant recipients. Early duplex ultrasonography should be considered for all high-risk patients, especially those with prolonged operative times or early signs of PGD or renal dysfunction. Our findings suggest that postoperative DVT is not a benign event and warrants prompt recognition and intervention. Furthermore, institutional standardization of perioperative anticoagulation protocols—including timing of reinitiation, target therapeutic ranges, and use of anti-Xa monitoring—may help mitigate variability and improve outcomes. Future multicenter studies are needed to determine optimal anticoagulation intensity and timing, balancing thrombosis prevention with bleeding risk in this fragile patient group.

This study has several limitations. Its retrospective design introduces potential for selection bias and unmeasured confounding. The analysis was performed at a single high-volume transplant center, which may limit generalizability to other institutions with different VV-ECMO practices or anticoagulation protocols. Duplex ultrasonography was not performed uniformly across all patients, raising the possibility of underdiagnosis of asymptomatic DVT. In addition, surveillance bias is possible, because VV-ECMO–bridged or otherwise higher-acuity patients may have been more likely to undergo diagnostic imaging, potentially increasing DVT detection independent of true incidence. Because the frequency of duplex ultrasonography was not available in a standardized manner, we were unable to quantify imaging intensity by subgroup, and this limitation should be considered when interpreting group comparisons. Additionally, information on long-term anticoagulation adherence and PE severity was limited. Because anticoagulation exposure was analyzed largely as a binary variable, our findings may be subject to time-dependent confounding and reverse causation if anticoagulation was initiated in response to evolving clinical suspicion or diagnosis of thromboembolic events; therefore, the observed association between anticoagulation and PE should not be interpreted as causal. Although case-level review in the VV-ECMO–bridged cohort confirmed that anticoagulation preceded PE diagnosis, residual time-dependent bias cannot be excluded in the overall cohort. Nonetheless, the large sample size, standardized institutional protocols, and long follow-up period strengthen the validity of our findings.

## Conclusion

In summary, post-transplant DVT represents a frequent and clinically significant complication among lung transplant recipients, particularly those bridged with VV-ECMO. DVT is associated with increased postoperative morbidity, prolonged hospitalization, and reduced survival. Preoperative VV-ECMO support identifies a subgroup with higher hemostatic complexity and greater susceptibility to thromboembolic events. Our findings highlight the need for vigilant DVT surveillance and optimization of perioperative anticoagulation strategies to improve post-transplant outcomes in this high-risk population.

## Supplementary Information

Below is the link to the electronic supplementary material.

Supplementary Material 1.

## Data Availability

The datasets generated and/or analyzed during the current study are available from the corresponding author on reasonable request.

## References

[1] Stevens SM, Woller SC, Baumann Kreuziger L, et al. Executive summary: antithrombotic therapy for VTE disease: second update of the CHEST guideline and expert panel report. Chest. 2021;160(6):2247–59. 10.1016/j.chest.2021.07.056. 34352279 10.1016/j.chest.2021.07.056

[2] Zheng M, Yousef I, Mamary AJ, et al. Venous thromboembolism in lung transplant recipients real world experience from a high volume center. J Heart Lung Transplant. 2021;40(10):1145–52. 10.1016/j.healun.2021.07.010. 34389222 10.1016/j.healun.2021.07.010

[3] Kurihara C, Manerikar A, Querrey M, et al. Clinical characteristics and outcomes of patients with COVID-19-associated acute respiratory distress syndrome who underwent lung transplant. JAMA. 2022;327(7):652–61. 10.1001/jama.2022.0204. 35085383 10.1001/jama.2022.0204 PMC8796055

[4] Toyoda T, Thomae BL, Kaiho T, et al. Impact of bridging veno-venous extracorporeal membrane oxygenation to COVID-19 lung transplantation. J Thorac Dis. 2024;16(7):4417–28. 10.21037/jtd-24-132. 39144296 10.21037/jtd-24-132 PMC11320280

[5] Toyoda T, Cerier EJ, Manerikar AJ, Kandula V, Bharat A, Kurihara C. Recipient, donor, and surgical factors leading to primary graft dysfunction after lung transplant. J Thorac Dis. 2023;15(2):399–409. 10.21037/jtd-22-974. 36910052 10.21037/jtd-22-974 PMC9992558

[6] Kurihara C, Manerikar A, Gao CA, et al. Outcomes after extracorporeal membrane oxygenation support in COVID-19 and non-COVID-19 patients. Artif Organs. 2022;46(4):688–96. 10.1111/aor.14090. 34694655 10.1111/aor.14090 PMC8653196

[7] Manerikar A, Watanabe S, Kandula V, et al. Indwelling central venous catheters drive bloodstream infection during veno-venous extracorporeal membrane oxygenation support. ASAIO J. 2022;68(6):859–64. 10.1097/MAT.0000000000001575. 34593682 10.1097/MAT.0000000000001575 PMC8958168

[8] Kurihara C, Manerikar A, Bharat A. Modern extracorporeal membrane oxygenation circuitry may obviate the need for continuous systemic anticoagulation. Ann Thorac Surg. 2022;113(1):375. 10.1016/j.athoracsur.2021.04.073. 33974891 10.1016/j.athoracsur.2021.04.073

[9] Krueger K, Schmutz A, Zieger B, Kalbhenn J. Venovenous extracorporeal membrane oxygenation with prophylactic subcutaneous anticoagulation only: an observational study in more than 60 patients. Artif Organs. 2017;41(2):186–92. 10.1111/aor.12737. 27256966 10.1111/aor.12737

[10] Parzy G, Daviet F, Puech B, et al. Venous thromboembolism events following venovenous extracorporeal membrane oxygenation for severe acute respiratory syndrome coronavirus 2 based on CT scans. Crit Care Med. 2020;48(10):e971–5. 10.1097/CCM.0000000000004504. 32618700 10.1097/CCM.0000000000004504 PMC7328443

[11] Martucci G, Panarello G, Occhipinti G, et al. Anticoagulation and transfusions management in veno-venous extracorporeal membrane oxygenation for acute respiratory distress syndrome: assessment of factors associated with transfusion requirements and mortality. J Intensive Care Med. 2019;34(8):630–9. 10.1177/0885066617706339. 28460592 10.1177/0885066617706339

[12] Munshi L, Walkey A, Goligher E, Pham T, Uleryk EM, Fan E. Venovenous extracorporeal membrane oxygenation for acute respiratory distress syndrome: a systematic review and meta-analysis. Lancet Respir Med. 2019;7(2):163–72. 10.1016/S2213-2600(18)30452-1. 30642776 10.1016/S2213-2600(18)30452-1

[13] Olson SR, Murphree CR, Zonies D, et al. Thrombosis and bleeding in extracorporeal membrane oxygenation (ECMO) without anticoagulation: a systematic review. ASAIO J. 2021;67(3):290–6. 10.1097/MAT.0000000000001230. 33627603 10.1097/MAT.0000000000001230 PMC8623470

[14] Bembea MM, Annich G, Rycus P, Oldenburg G, Berkowitz I, Pronovost P. Variability in anticoagulation management of patients on extracorporeal membrane oxygenation: an international survey. Pediatr Crit Care Med. 2013;14(2):e77-84. 10.1097/PCC.0b013e31827127e4. 23287906 10.1097/PCC.0b013e31827127e4 PMC3567253

[15] Tonna JE, Abrams D, Brodie D, et al. Management of adult patients supported with venovenous extracorporeal membrane oxygenation (VV ECMO): guideline from the extracorporeal life support organization (ELSO). ASAIO J. 2021;67(6):601–10. 10.1097/MAT.0000000000001432. 33965970 10.1097/MAT.0000000000001432 PMC8315725

[16] Kurihara C, Walter JM, Karim A, et al. Feasibility of venovenous extracorporeal membrane oxygenation without systemic anticoagulation. Ann Thorac Surg. 2020;110(4):1209–15. 10.1016/j.athoracsur.2020.02.011. 32173339 10.1016/j.athoracsur.2020.02.011 PMC7486253

[17] Fan E, Del Sorbo L, Goligher EC, et al. An official American thoracic society/European society of intensive care medicine/society of critical care medicine clinical practice guideline: mechanical ventilation in adult patients with acute respiratory distress syndrome. Am J Respir Crit Care Med. 2017;195(9):1253–63. 10.1164/rccm.201703-0548ST. 28459336 10.1164/rccm.201703-0548ST

[18] Badulak J, Antonini MV, Stead CM, et al. Extracorporeal membrane oxygenation for COVID-19: updated 2021 guidelines from the extracorporeal life support organization. ASAIO J. 2021;67(5):485–95. 10.1097/MAT.0000000000001422. 33657573 10.1097/MAT.0000000000001422 PMC8078022

[19] Chang A, Miyashita Y, Thomae BL, Kamar A, Kaiho T, Kurihara C. Outcomes of venovenous-extracorporeal membrane oxygenation bridging in lung transplant recipients with panel reactive antibody positivity. J Artif Organs. 2026;29(1):2. 10.1007/s10047-025-01539-2. 10.1007/s10047-025-01539-2 PMC12615553 41233608

[20] Kaniuk JK, Miyashita Y, Kamar A, Kaiho T, Schipma MJ, Kurihara C. Impact of pre-transplant veno-venous extracorporeal membrane oxygenation on post-lung transplant infections. J Artif Organs. 2026;29(1):6. 10.1007/s10047-025-01529-4. 10.1007/s10047-025-01529-4 PMC12618338 41239051

[21] Kurihara C, Miyashita Y, Kaiho T, Yamanouchi D. Feasibility of anticoagulation-free peripheral veno-arterial extracorporeal membrane oxygenation in re-do lung transplantation. J Artif Organs. 2026;29(1):15. 10.1007/s10047-025-01541-8. 10.1007/s10047-025-01541-8 PMC12705707 41396406

[22] Kim NT, Miyashita Y, Kaihou T, Kim JT, Kurihara C. Risk factors and perioperative complications associated with deep venous thrombosis and pulmonary embolism after lung transplantation. J Thorac Dis. 2025;17(11):9342–56. 10.21037/jtd-2025-1294. 41376962 10.21037/jtd-2025-1294 PMC12688489

[23] Snell GI, Yusen RD, Weill D, et al. Report of the ISHLT working group on primary lung graft dysfunction, part i: definition and grading-a 2016 consensus group statement of the international society for heart and lung transplantation. J Heart Lung Transplant. 2017;36(10):1097–103. 10.1016/j.healun.2017.07.021. 28942784 10.1016/j.healun.2017.07.021

[24] Verleden GM, Glanville AR, Lease ED, et al. Chronic lung allograft dysfunction: definition, diagnostic criteria, and approaches to treatment-A consensus report from the pulmonary council of the ISHLT. J Heart Lung Transplant. 2019;38(5):493–503. 10.1016/j.healun.2019.03.009. 30962148 10.1016/j.healun.2019.03.009

[25] Bellomo R, Ronco C, Kellum JA, Mehta RL, Palevsky P. ADQI workgroup. Acute renal failure–definition, outcome measures, animal models, fluid therapy and information technology needs: the second international consensus conference of the acute dialysis quality initiative (ADQI) group. Crit Care. 2004;8(4):R204. 15312219 10.1186/cc2872 PMC522841

[26] Li LJ, Xu HY, Wang XW, et al. Impact of delayed veno-venous extracorporeal membrane oxygenation weaning on postoperative rehabilitation of lung transplantation: a single-center comparative study. J Artif Organs. 2023;26(4):303–8. 10.1007/s10047-022-01376-7. 36482123 10.1007/s10047-022-01376-7

[27] Hashimoto K, Hoetzenecker K, Yeung JC, et al. Intraoperative extracorporeal support during lung transplantation in patients bridged with venovenous extracorporeal membrane oxygenation. J Heart Lung Transplant. 2018;37(12):1418–24. 10.1016/j.healun.2018.07.003. 30193763 10.1016/j.healun.2018.07.003

[28] Rinieri P, Peillon C, Bessou JP, et al. National review of use of extracorporeal membrane oxygenation as respiratory support in thoracic surgery excluding lung transplantation. Eur J Cardiothorac Surg. 2015;47(1):87–94. 10.1093/ejcts/ezu127. 24659317 10.1093/ejcts/ezu127

[29] Xia Y, Ragalie W, Yang EH, et al. Venoarterial versus venovenous extracorporeal membrane oxygenation as bridge to lung transplantation. Ann Thorac Surg. 2022;114(6):2080–6. 10.1016/j.athoracsur.2021.11.016. 34906571 10.1016/j.athoracsur.2021.11.016

[30] Iannattone PA, Yang SS, Koolian M, Wong EG, Lipes J. Incidence of venous thromboembolism in adults receiving extracorporeal membrane oxygenation: a systematic review. ASAIO J. 2022;68(12):1523–8. 10.1097/MAT.0000000000001694. 36469448 10.1097/MAT.0000000000001694

[31] Zhu Y, Lan MJ, Liang JS, et al. Assessing venous thrombotic risks in extracorporeal membrane oxygenation-supported patients: a systematic review and meta-analysis. Clin Appl Thromb Hemost Jan-Dec. 2024;30:10760296241279292. 10.1177/10760296241279293. 10.1177/10760296241279293 PMC11388296 39246243

[32] Menaker J, Tabatabai A, Rector R, et al. Incidence of cannula-associated deep vein thrombosis after veno-venous extracorporeal membrane oxygenation. ASAIO J Sep/Oct. 2017;63(5):588–91. 10.1097/MAT.0000000000000539. 10.1097/MAT.0000000000000539 28857905

