# Supplementary material for: Anticoagulation optimization and clinical impact of post-transplant deep vein thrombosis: clinical impact and a VV-ECMO subgroup analysis
Source: J Artif Organs. 2026 May 5;29(2):28. doi: 10.1007/s10047-026-01554-x (PMC13144232; doi:10.1007/s10047-026-01554-x)
Supplement: Supplementary file 1 — Supplementary Material 1. [file 10047_2026_1554_MOESM1_ESM.pdf]

Supplemental file for

Anticoagulation Optimization and Clinical Impact of Post-Transplant Deep Vein Thrombosis in ECMO-Bridged Lung Transplant Recipients

Chitaru Kurihara, Yudai Miyashita, Taisuke Kaiho, Dai Yamanouchi

This file contains

Supplementary Table1-4

Supplementary Figure 1

Supplementary Table 1. Characteristics of Patients

Variable	DVT (n=240)	No DVT (n=262)	*P* value
**Pre-operative Characteristics**			
Age, years	62.0 (55.8, 68.0)	62.0 (52.0, 68.0)	0.2
Sex			
Male	142/240 (59.2%)	144/262 (55.0%)	0.37
Female	98/240 (40.8%)	118/262 (45.0%)	0.37
BMI, kg/m2	26.6 (22.2, 29.6)	26.4 (22.3, 29.4)	0.7
BSA, m2	1.9 (1.7, 2.1)	1.8 (1.7, 2.0)	0.26
Smoking history			
Yes	122/240 (50.8%)	140/262 (53.4%)	0.59
No	118/240 (49.2%)	122/262 (46.6%)	0.59
Hypertension	141/240 (58.8%)	150/262 (57.3%)	0.79
Diabetes	74/240 (30.8%)	74/262 (28.2%)	0.56
CKD	26/240 (10.8%)	20/262 (7.6%)	0.22
Dialysis	13/240 (5.4%)	10/262 (3.8%)	0.4
On the waiting list (days)	9.0 (4.0, 32.0)	8.0 (4.0, 22.0)	0.08
ECMO bridge	31/240 (12.9%)	22/262 (8.4%)	0.11
Etiology			
ILD	115/240 (47.9%)	128/262 (48.9%)	0.86
COPD	49/240 (20.4%)	47/262 (17.9%)	0.5
PAH	12/240 (5.0%)	27/262 (10.3%)	0.03
Others	64/240 (26.7%)	60/262 (22.9%)	0.35
Laboratory			
Hemoglobin, g/dL	11.9 (9.6, 13.2)	11.9 (10.3, 13.8)	0.14
Platelets, 1,000/mm3	232.0 (185.0, 298.0)	242.5 (191.5, 297.0)	0.33
Creatinine, mg/dL	0.8 (0.6, 0.9)	0.8 (0.6, 0.9)	0.12
INR	1.0 (1.0, 1.1)	1.0 (1.0, 1.1)	0.96
PTT	30.5 (27.8, 33.7)	30.5 (28.1, 33.5)	0.88
PRA	0.0 (0.0, 1.0)	0.0 (0.0, 1.0)	0.61
**Intra-operative outcomes**			
Bilateral	156/240 (65.0%)	168/262 (64.1%)	0.85
Operative time (hours)	5.6 (4.6, 7.4)	5.1 (3.9, 6.6)	< .0001
Intra-op blood transfusion; pRBC	1.0 (0.0, 3.0)	0.0 (0.0, 2.0)	0.1
Intra-op blood transfusion; FFP	0.0 (0.0, 0.0)	0.0 (0.0, 0.0)	0.18
Intra-op blood transfusion; Plt	0.0 (0.0, 0.0)	0.0 (0.0, 0.0)	0.12
VA ECMO use	151/240 (62.9%)	157/262 (59.9%)	0.52
VA ECMO time (hours)	2.2 (0.0, 3.0)	2.1 (0.0, 2.8)	0.15

**Post-operative outcomes**			
de novo DSA	29/239 (12.1%)	45/262 (17.2%)	0.13
CVA	9/240 (3.8%)	8/262 (3.1%)	0.81
Bowel Ischemia	4/240 (1.7%)	2/262 (0.8%)	0.43
Digital Ischemia	6/240 (2.5%)	5/262 (1.9%)	0.76
Days of DVT after lung txplt	22.0 (7.5, 81.5)	NA	
PE	54/240 (22.5%)	13/262 (5.0%)	<.0001
Days of PE after lung txplt	50.0 (12.0, 93.0)	29.0 (13.0, 47.0)	0.48
Anticoagulation	101/240 (42.1%)	74/262 (28.2%)	< .0001
AKI	127/240 (52.9%)	108/262 (41.2%)	0.009
PGD grade3	44/240 (18.3%)	28/262 (10.7%)	0.02
Dialysis	13/240 (5.4%)	10/262 (3.8%)	0.4
HD after discharge	48/240 (20.0%)	13/262 (5.0%)	< .0001
Post transplant ventilator	2.0 (1.0, 4.0)	2.0 (1.0, 3.0)	0.25
Hospital stay	21.0 (12.0, 37.0)	14.0 (11.0, 24.0)	< .0001
post ECMO use	40/240 (16.7%)	23/262 (8.8%)	0.01

Continuous data are shown as medians and and interquartile ranges (Q1-Q3) for days. CLAD, chronic lung allograft dysfunction; AKI, acute kidney injury; BMI, body mass index; BSA, body surface area; ECMO, extracorporeal membrane oxygenation; LAS, lung allocation score; COPD, chronic obstructive pulmonary disease; CPFE, combined pulmonary fibrosis and emphysema; ILD, interstitial lung disease; ARDS, acute respiratory distress syndome; COVID-19, coronavirus disease 2019; PAH, pulmonary arterial hypertension; WBC, white blood cell; BUN, blood urea nitrogen; INR, international normalized ratio; PRA, panel reactive antibody

Supplementary Table 2. Patient characteristics in the DVT cohort according preoperative VV-ECMO use

Variable	VV-ECMO (n=31)	No VV-ECMO (n=209)	*P* value
**Pre-operative Characteristics**			
Age, years	53.0 (43.5, 58.5)	63.0 (57.0, 68.0)	<0.001
Sex			
Male	16/31 (51.6%)	126/209 (60.3%)	0.434
Female	15/31 (48.4%)	83/209 (39.7%)	0.434
BMI, kg/m2	26.1 (22.5, 28.6)	26.6 (22.2, 29.7)	0.982
BSA, m2	1.9 (1.7, 2.1)	1.9 (1.7, 2.1)	0.73
Smoking history			<0.001
Yes	5/31 (16.1%)	117/209 (56.0%)	
No	26/31 (83.9%)	92/209 (44.0%)	
Hypertension	15/31 (48.4%)	126/209 (60.3%)	0.24
Diabetes	6/31 (19.4%)	68/209 (32.5%)	0.15
CKD	1/31 (3.2%)	25/209 (12.0%)	0.22
Dialysis	6/31 (19.4%)	7/209 (3.3%)	0.00
On the waiting list (days)	7.0 (4.0, 24.5)	9.0 (4.0, 32.0)	0.34
Preoperative VV-ECMO			
Etiology			
ILD	9/31 (29.0%)	106/209 (50.7%)	0.033
COPD	0/31 (0.0%)	49/209 (23.4%)	<0.001
PAH	1/31 (3.2%)	11/209 (5.3%)	1.00
Others	21/31 (67.7%)	43/209 (20.6%)	<0.001
Laboratory			
Hemoglobin, g/dL	7.5 (7.2, 8.5)	12.2 (10.6, 13.6)	<0.001
Platelets, 1,000/mm3	151.0 (103.0, 197.5)	240.0 (199.2, 305.0)	<0.001
Creatinine, mg/dL	0.6 (0.4, 0.8)	0.8 (0.7, 1.0)	<0.001
INR	1.2 (1.1, 1.3)	1.0 (1.0, 1.1)	<0.001
PTT	34.3 (29.9, 38.2)	30.3 (27.6, 33.0)	<0.001
PRA	1.0 (0.0, 1.0)	0.0 (0.0, 1.0)	0.01
**Intra-operative outcomes**			
Bilateral	30/31 (96.8%)	126/209 (60.3%)	<0.001
Operative time (hours)	8.2 (6.7, 9.6)	5.4 (4.3, 7.1)	<0.001
Intra-op blood transfusion; pRBC	7.0 (5.0, 11.5)	0.0 (0.0, 2.0)	<0.001
Intra-op blood transfusion; FFP	3.0 (1.5, 6.0)	0.0 (0.0, 0.0)	<0.001
Intra-op blood transfusion; Plt	2.0 (1.0, 3.5)	0.0 (0.0, 0.0)	<0.001
VA ECMO use	30/31 (96.8%)	121/209 (57.9%)	<0.001
**Post-operative outcomes**			

de novo DSA	7/31 (22.6%)	32/208 (15.4%)	0.30
CVA	0/31 (0.0%)	9/209 (4.3%)	0.61
Bowel Ischemia	0/31 (0.0%)	4/209 (1.9%)	1.00
Digital Ischemia	2/31 (6.5%)	4/209 (1.9%)	0.17
Days of DVT after lung txplt	18.0 (6.0, 51.0)	22.0 (8.8, 85.8)	0.13
PE	7/31 (22.6%)	45/209 (21.5%)	1.00
Days of PE after lung txplt	56.0 (5.5, 70.0)	44.0 (13.0, 99.0)	0.41
Anticoagulation	19/31 (61.3%)	82/209 (39.2%)	0.03
AKI	20/31 (64.5%)	107/209 (51.2%)	0.182
PGD grade3	14/31 (45.2%)	30/209 (14.4%)	<0.001
Dialysis	6/31 (19.4%)	7/209 (3.3%)	0.00
HD after discharge	10/31 (32.3%)	38/207 (18.4%)	0.09
Post transplant ventilator	3.0 (1.5, 13.5)	2.0 (1.0, 3.0)	0.01
Hospital stay	35.0 (25.0, 44.0)	19.0 (12.0, 34.0)	<0.001
post ECMO use	21/31 (67.7%)	19/209 (9.1%)	<0.001

Continuous data are shown as medians and and interquartile ranges (Q1-Q3) for days. AKI, acute kidney injury; ARDS, acute respiratory distress syndrome; BSA, body surface area; BMI, body mass index; BUN, blood urea nitrogen; CKD, chronic kidney disease; CLAD, chronic lung allograft dysfunction; COPD, chronic obstructive pulmonary disease; CPFE, combined pulmonary fibrosis and emphysema; CVA, cerebrovascular accident; DSA, donor-specific antibody; DVT, deep vein thrombosis; ECMO, extracorporeal membrane oxygenation; FFP, fresh frozen plasma; HD, hemodialysis; ICU, intensive care unit; ILD, interstitial lung disease; INR, international normalized ratio; LAS, lung allocation score; PAH, pulmonary arterial hypertension; PE, pulmonary embolism; PGD, primary graft dysfunction; Plt, platelets; PRA, panel reactive antibody; pRBC, packed red blood cells; PTT, partial thromboplastin time; VA-ECMO, venoarterial extracorporeal membrane oxygenation; VV-ECMO, venovenous extracorporeal membrane oxygenation; WBC, white blood cell.

Supplementary Table 3. DVT locations within 14 days post-transplant, stratified by preoperative VV-ECMO use, with anticoagulation by location

Location	Preoperative VV-ECMO use			Anticoagulation		
	VV-ECMO (n=15)	No VV-ECMO (n=81)	p-value	VV-ECMO	No VV-ECMO	p-value
UE	8 (53.3%)	19 (23.5%)	0.03	6/8 (75.0%)	15/19 (78.9%)	1.00
UE alone	3 (20.0%)	6 (7.4%)	0.15	2/3 (66.7%)	3/6 (50.0%)	1.00
UE + LE below knee	2 (13.3%)	2 (2.5%)	0.11	2/2 (100.0%)	1/2 (50.0%)	1.00
UE + Neck	3 (20.0%)	8 (9.9%)	0.37	2/3 (66.7%)	8/8 (100.0%)	0.27
Neck + UE + LE above knee	0 (0.0%)	1 (1.2%)	1.00	NA	1/1 (100.0%)	NA
Neck+UE+LE below knee	0 (0.0%)	1 (1.2%)	1.00	NA	1/1 (100.0%)	NA
Neck + UE + LE below knee + LE above knee	0 (0.0%)	1 (1.2%)	1.00	NA	1/1 (100.0%)	NA
LE above knee	3 (20.0%)	19 (23.5%)	1.00	3/3 (100.0%)	13/19 (68.4%)	0.53
LE above knee alone	2 (13.3%)	10 (12.3%)	1.00	2/2 (100.0%)	4/10 (40.0%)	0.45
LE above & below knee	1 (6.7%)	7 (8.6%)	1.00	1/1 (100.0%)	7/7 (100.0%)	1.00
Neck + UE + LE above knee	0 (0.0%)	1 (1.2%)	1.00	NA	1/1 (100.0%)	NA
Neck + UE + LE below knee + LE above knee	0 (0.0%)	1 (1.2%)	1.00	NA	1/1 (100.0%)	NA
LE below knee	6 (40.0%)	35 (43.2%)	1.00	6/6 (100.0%)	26/35 (54.3%)	0.31
LE below knee alone	3 (20.0%)	19 (23.5%)	1.00	3/3 (100.0%)	13/19 (74.3%)	0.53
LE above & below knee	1 (6.7%)	7 (8.6%)	1.00	1/1 (100.0%)	7/7 (100.0%)	1.00
UE + LE below knee	2 (13.3%)	2 (2.5%)	0.11	2/2 (100.0%)	1/2 (50.0%)	1.00
Neck + LE below knee	0 (0.0%)	5 (6.2%)	1.00	NA	3/5 (60.0%)	NA
Neck+UE+LE below knee	0 (0.0%)	1 (1.2%)	1.00	NA	1/1 (100.0%)	NA
Neck + UE + LE below knee + LE above knee	0 (0.0%)	1 (1.2%)	1.00	NA	1/1 (100.0%)	NA

Neck vein	4 (26.7%)	37 (45.7%)	0.26	2/4 (50.0%)	26/37 (70.3%)	0.58
Neck vein alone	1 (6.7%)	21 (25.9%)	0.18	0/1 (0.0%)	12/21 (57.1%)	0.45
UE + Neck	3 (20.0%)	8 (9.9%)	0.37	2/3 (66.7%)	8/8 (100.0%)	0.27
Neck + UE + LE above knee	0 (0.0%)	1 (1.2%)	1.00	NA	1/1 (100.0%)	NA
Neck + LE below knee	0 (0.0%)	5 (6.2%)	1.00	NA	3/5 (60.0%)	NA
Neck+UE+LE below knee	0 (0.0%)	1 (1.2%)	1.00	NA	1/1 (100.0%)	NA
Neck + UE + LE below knee + LE above knee	0 (0.0%)	1 (1.2%)	1.00	NA	1/1 (100.0%)	NA

DVT, deep vein thrombosis; ECMO, extracorporeal membrane oxygenation; LE, lower extremity; UE, upper extremity; NA, not applicable; VV-ECMO, venovenous extracorporeal membrane oxygenation. Percentages are based on all DVT cases within each group. Because subcategories are not mutually exclusive, parent and child percentages are not additive. everal entries are duplicate listings across anatomical blocks. P values are from two-sided Fisher's exact tests.

Supplementary Table 4. Case-level anticoagulation timing relative to pulmonary embolism diagnosis in the VV cohort

Case No.	DVT	Anticoagulation	Anticoagulation prior to PE	Days on anticoagulation from LTx	Day of PE diagnosis from LTx
1	Yes	Yes	Yes	15	60
2	Yes	Yes	Yes	3	4
3	Yes	Yes	Yes	0	80
4	Yes	No	No		173
5	Yes	No	No		91
6	Yes	Yes	Yes	1	56
7	Yes	Yes	Yes	4	7
8	No	No	No		6

Time variables are expressed as days from lung transplantation (LTx). “Anticoagulation” indicates whether the patient received systemic anticoagulation at any time. “Anticoagulation prior to PE” denotes anticoagulation exposure before the day of PE diagnosis. “Days on anticoagulation from LTx” represents the post transplant of days on anticoagulation; blank cells indicate no anticoagulation prior to PE. “Day of PE diagnosis from LTx” indicates the post-transplant day on which PE was diagnosed. No patients initiated anticoagulation after PE diagnosis in this cohort; therefore, sensitivity analyses addressing post-PE anticoagulation yielded results identical to the main analysis.

Supplementary Table 5. Anticoagulation Use According to preoperative VV-ECMO Status, Presence of DVT and PE

ECMO	VV-ECMO				no VV-ECMO			
DVT	DVT		no DVT		DVT		no DVT	
PE	PE	no PE	PE	no PE	PE	no PE	PE	no PE
anticoagulation	3/6 (50.0%)	16/25 (64.0%)	0/0 (0.0%)	10/21 (47.6%)	17/25 (68.0%)	65/184 (35.3%)	7/13 (53.8%)	53/222 (23.9%)

DVT, deep vein thrombosis; ECMO, extracorporeal membrane oxygenation; PE, pulmonary embolism; VA-ECMO, venoarterial extracorporeal membrane oxygenation; VV-ECMO, venovenous extracorporeal membrane oxygenation. Anticoagulation use is expressed as the number and percentage of patients receiving anticoagulation therapy, stratified by preoperative VV-ECMO or intraoperative VA-ECMO status, and by the presence or absence of DVT and PE.

Supplementary Figure 1

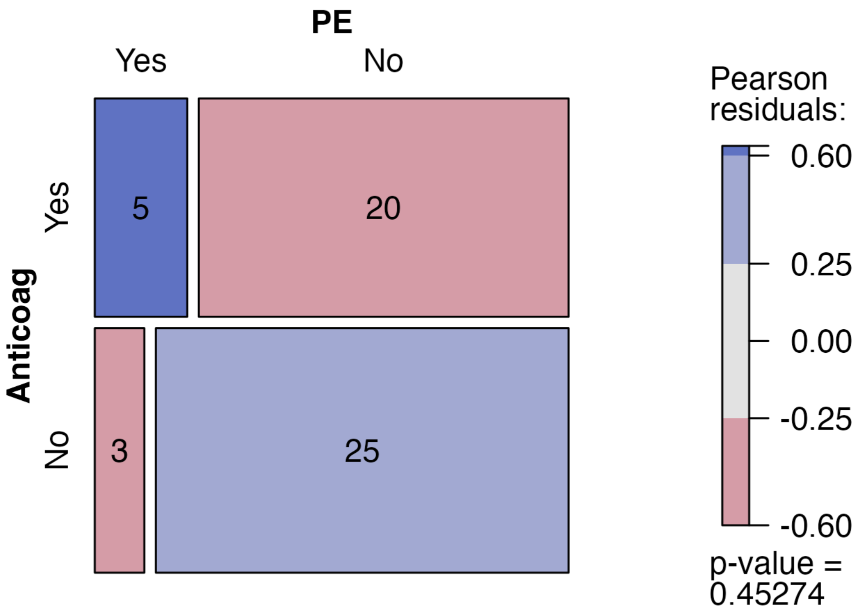

Supplementary Figure 1. Association between anticoagulation use and PE in patients with DVT.

Mosaic plot depicting the distribution of pulmonary embolism (PE) by anticoagulation status among patients bridged with venovenous extracorporeal membrane oxygenation (VV-ECMO) prior to lung transplantation. Each rectangle area is proportional to the corresponding cell count; numbers denote observed frequencies. Cell shading reflects Pearson residuals from the independence model, with blue indicating higher-than-expected and red indicating lower-than-expected counts. The odds ratio (OR) with 95% confidence interval (CI) and Fisher's exact test p-value are shown.

Supplementary Figure 2

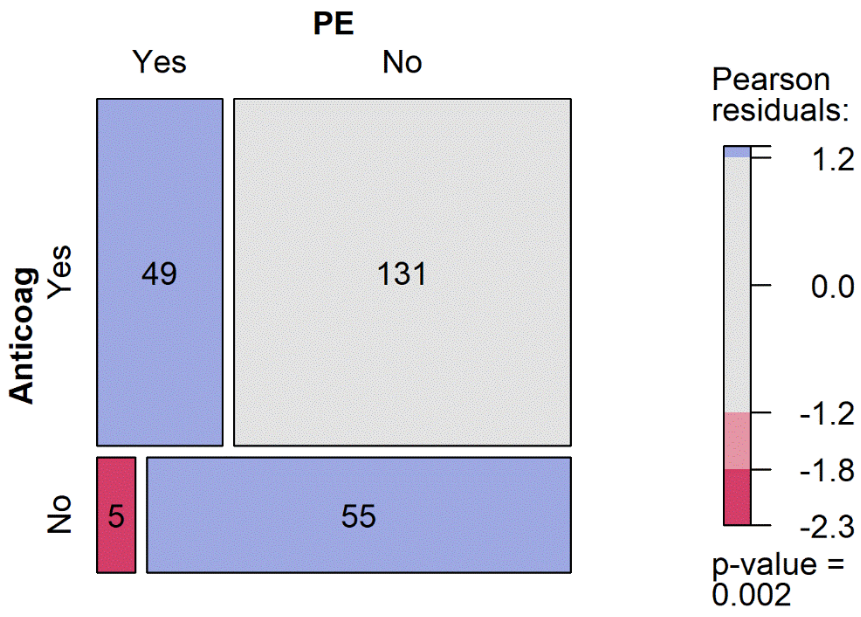

Supplementary Figure 2. Association between anticoagulation use and PE in patients with DVT.

Mosaic plot demonstrating the relationship between anticoagulation and PE among patients who developed DVT.

Supplementary Figure 3

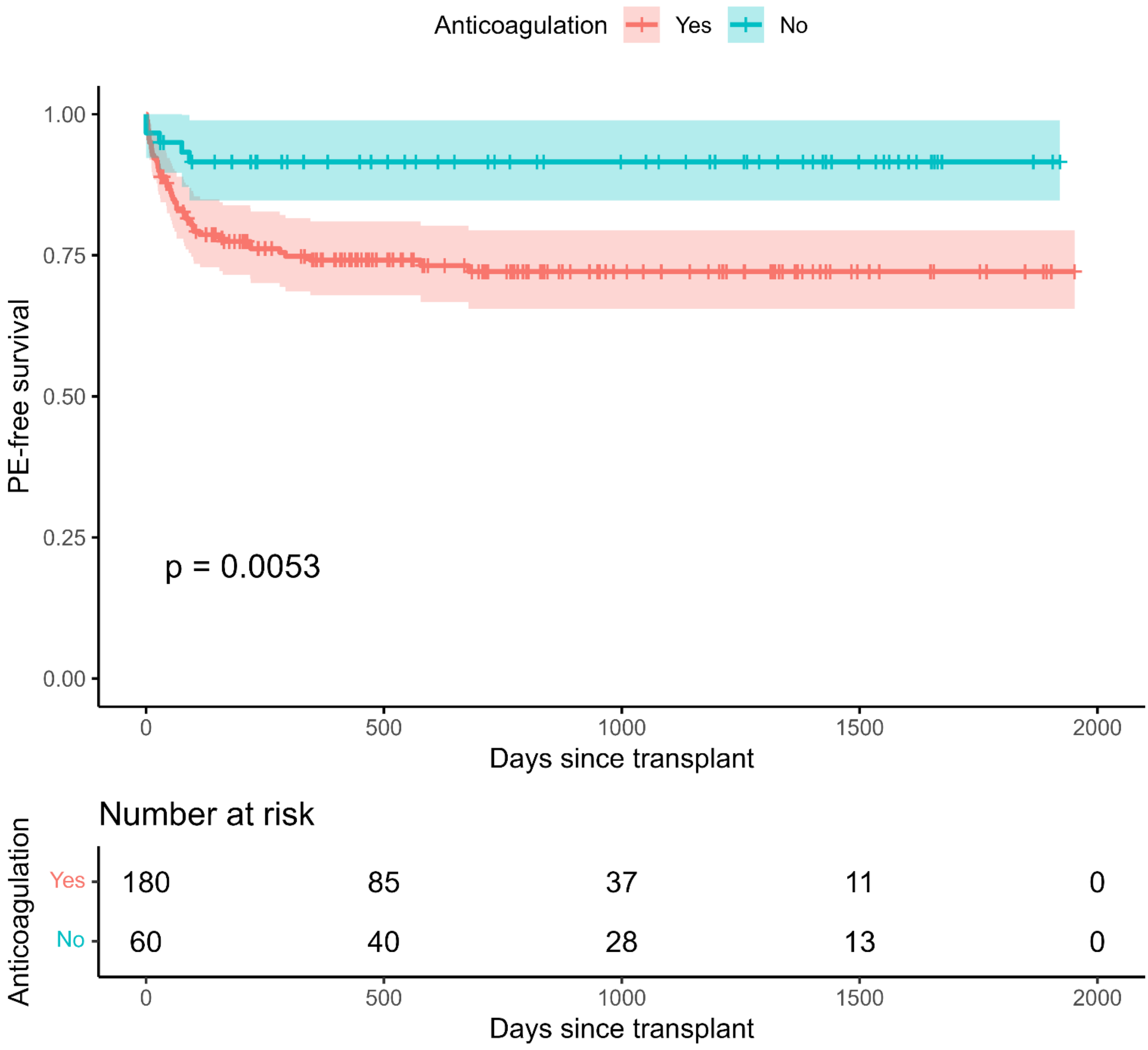

Supplementary Figure 3. Pulmonary embolism–free survival according to anticoagulation use.

Kaplan–Meier curves showing PE-free survival among patients with DVT, stratified by anticoagulation status.

Supplementary Figure 4

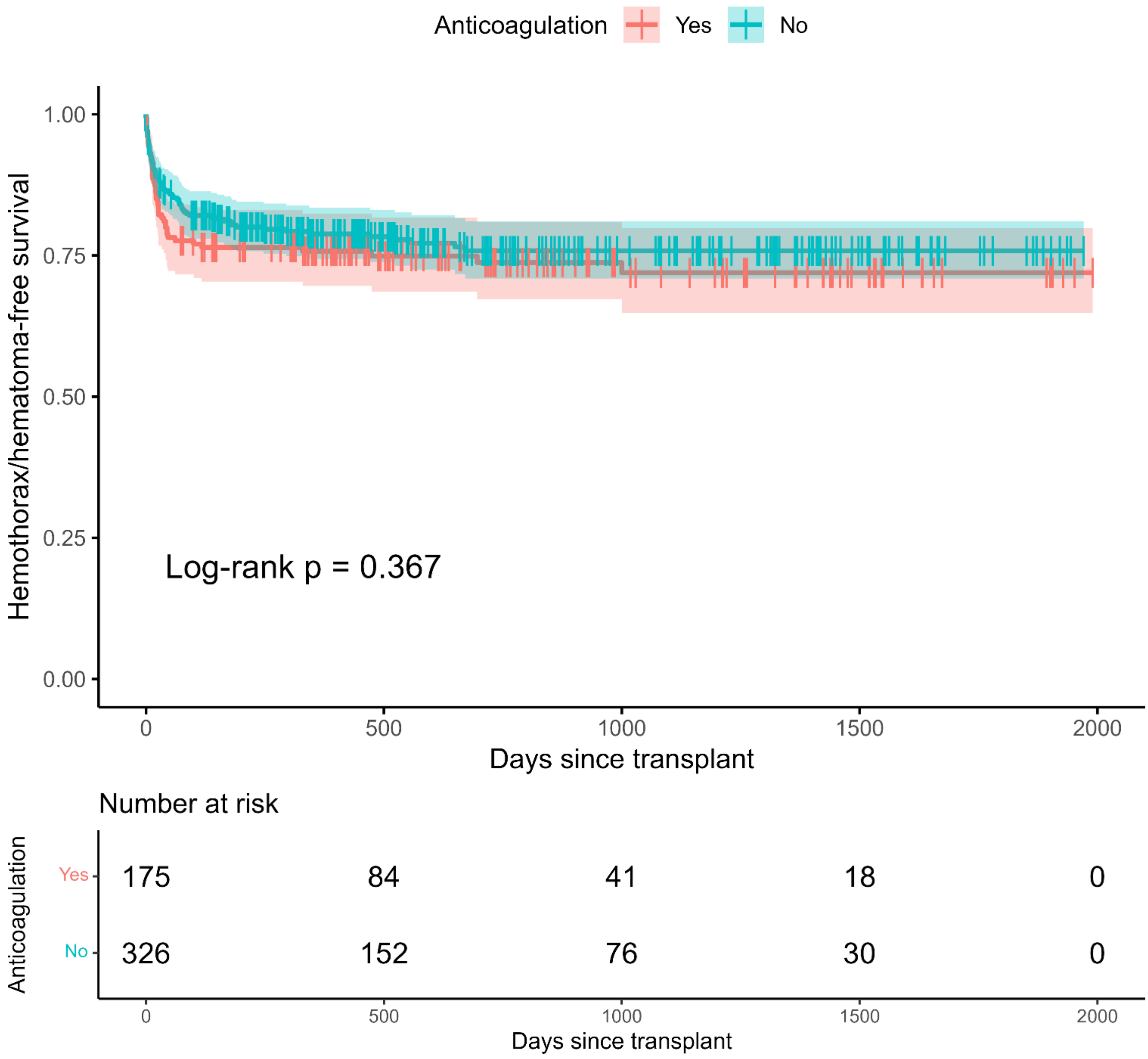

Supplementary Figure 4. Hemothorax/hematoma-free survival according to anticoagulation use.

Kaplan–Meier curves comparing hemothorax or hematoma-free survival between patients with and without anticoagulation therapy.
